# Growth arrest and DNA damage-inducible 45: a new player on inflammatory diseases

**DOI:** 10.3389/fimmu.2025.1513069

**Published:** 2025-02-27

**Authors:** Yanmei Ma, Md Munnaf Hossen, Jennifer Jin Huang, Zhihua Yin, Jing Du, Zhizhong Ye, Miaoyu Zeng, Zhong Huang

**Affiliations:** ^1^ Rheumatology Research Institute, Shenzhen Futian Hospital for Rheumatic Diseases, Shenzhen, China; ^2^ Department of Immunology, Biological Therapy Institute, Guangdong Provincial Key Laboratory of Regional Immunity and Diseases, Health Science Center, Shenzhen University, Shenzhen, China; ^3^ Joint Research Laboratory for Rheumatology of Shenzhen University Health Science Center and Shenzhen Futian Hospital for Rheumatic Diseases, Shenzhen, China; ^4^ Department of Chemistry and Biochemistry, University of Oklahoma, Norman, OK, United States; ^5^ Department of Laboratory Medicine, Peking University Shenzhen Hospital, Shenzhen, China

**Keywords:** GADD45, immunoregulation, auto-immunoregulation, inflammatory diseases, autoimmune diseases

## Abstract

Growth arrest and DNA damage-inducible 45 (GADD45) proteins are critical stress sensors rapidly induced in response to genotoxic/physiological stress and regulate many cellular functions. Even though the primary function of the proteins is to block the cell cycle, inhibit cell proliferation, promote cell apoptosis, and repair DNA damage to cope with the damage caused by internal and external stress on the body, evidence has shown that GADD45 also has the function to modulate innate and adaptive immunity and plays a broader role in inflammatory and autoimmune diseases. In this review, we focus on the immunomodulatory role of GADD45 in inflammatory and autoimmune diseases. First, we describe the regulatory factors that affect the expression of GADD45. Then, we introduce its immunoregulatory roles on immune cells and the critical signaling pathways mediated by GADD45. Finally, we discuss its immunomodulatory effects in various inflammatory and autoimmune diseases.

## 1 Introduction

In mammals, GADD45 is a gene family consisting of GADD45α, GADD45β, and GADD45γ, localized to three distinct chromosomes (chr 1, 19, and 9 for GADD45α, GADD45β, and GADD45γ, respectively) (1). GADD45 proteins are small (18 kD), evolutionarily conserved that are highly homologous to each other (55–57% overall identity at the amino acid level), highly acidic (pH ¼ 4.0–4.2), low abundance in normal cells, and localize in both nucleus and cytoplasm (2–7). The first GADD45 gene was identified in Chinese hamster (CHO) cells based on increased expression after growth cessation signals or treatment with DNA-damaging agents (8, 9). It was, therefore, given the abbreviation Growth Arrest and DNA Damage (GADD) as its name. This gene is renamed as GADD45α. Another gene of the GADD45 family, GADD45β (designated initially as MyD118), was identified as a primary response gene transiently induced by IL-6 in myeloid leukemia cell lines (2). GADD45γ was first described in mice as the ortholog of the human CR6 gene encoding an acute phase response protein expressed upon interleukin-2 stimulation (10).

The GADD45 gene has been proven to be expressed in various tissues, including the heart, brain, lungs, kidney, spleen, skeletal muscle, ovary, and testis (3, 11), as well as in drosophila (12, 13).

Due to the lack of enzyme activity, the physiological function of GADD45 proteins depends on protein-protein interactions with their partner proteins, which include proliferating cell nuclear antigen (PCNA), cell division cycle 2 kinase (cdc2)/cyclinB1, cyclin-dependent kinase 1(cdk1), cyclin-dependent kinase inhibitor 1A (p21), and mitogen-activated protein kinase kinase kinase 4 (MEKK4), p38 mitogen-activated protein kinase (P38 MAPK), and c-Jun N-terminal kinases (JNKs) (5, 14). As sensors of bodily and environmental damage, GADD45 family proteins play a critical role in various cellular functions and regulate diverse cellular effects (15), such as cell cycle arrest (4), DNA demethylation and repair (16), cell survival (17, 18), maintenance of genomic stability (19), and apoptosis (20, 21) in response to environmental and physiological stress, as well as having a role in development and carcinogenesis (22, 23). GADD45 responds to various immune signaling pathways induced by cytokines and T-cell receptors (TCR) and is involved in regulating intrinsic and acquired immunity (15). Notably, an increasing number of studies have confirmed the regulatory role of GADD45 in immunity (24–27). Studies in disease models and clinical trial specimens have implicated that GADD45 is involved in the pathogenesis of inflammatory autoimmune diseases (28, 29). In this review, we will focus on the immunomodulatory role of GADD45 in inflammatory and autoimmune diseases.

Notably, an increasing number of studies have confirmed the regulatory role of GADD45 in immunity (24–26). Studies in disease models and clinical trial specimens have indicated that GADD45 is involved in the pathogenesis of inflammatory autoimmune diseases (28, 29). In this review, we focus on the immunomodulatory role of GADD45 in inflammatory and autoimmune diseases.

## 2 The inducers of GADD45

GADD45 proteins are typical signaling proteins. They are small and rapidly regulated at both transcriptional and posttranscriptional levels, playing various roles in mediating stress signaling and growth regulation. Many factors, such as radiation, chemicals, inflammatory cytokines, and transcription factors, can trigger GADD45 expression to produce inflammatory and/or immunomodulatory effects. Each GADD45 gene has a distinctive expressional pattern in response to specific stressors.

### 2.1 Environmental stresses

#### 2.1.1 Radiation

Both mRNA and protein of GADD45α are induced by ionizing radiation (IR) in a human myeloid leukemia cell line (ML-1 cells) and a human colon adenocarcinoma cell line (RKO cells) (30). X-rays and γ irradiation have also been reported to induce GADD45α (17, 31, 32). All three GADD45 proteins were rapidly induced after treating RKO cells with UV, displaying somewhat different expression kinetics (4). In addition, expression of GADD45α and GADD45β was observed in ML-1 cells (17, 30) and Bone marrow (BM) cells (21).

#### 2.1.2 Chemical reagents

The expression of GADD45α, β, and γ genes in ML-1 cells can be highly induced by methyl methane sulfonate (MMS) (17). GADD45α and GADD45β but not GADD45γ were induced rapidly in M1 myeloblastic leukemia cells following treatment with MMS (3, 4). MMS-induced expressions of GADD45α in Chinese hamster ovary (CHO) and RKO cells were also reported (9, 33). In addition to MMS, other chemicals can also induce GADD45; for example, alkylating agent methyl malonyl sulfonate can induce GADD45α expression in ML-1 cells (30), and carbon tetrachloride (CCl4) can induce GADD45β (34).

#### 2.1.3 Other environmental factors

Following serum starvation of the M1 myeloblastic leukemia cells for 48h and stimulation with serum, the level of GADD45α mRNA was rapidly increased. Also, the levels of GADD45β and GADD45γ mRNAs transiently increased in BALB/c 3T3 cells after serum stimulation (3, 35). In addition, other environmental factors, such as H_2_O_2_, anisomycin (17), heat shock (36), heavy metals (37, 38), sodium arsenite (34), Arsenic (39–44), hypoxia (45–47), low pH (48), hyperosmotic stress (49–52), cisplatin (53–55), ethanol (56), low-frequency electromagnetic fields (57), peroxynitrite free radicals (58), cigarette smoke condensate (59, 60), mitomycin C (55), metal nanoparticles (61) have shown to induce the expression of GADD45.

### 2.2 Inflammatory factors

Evidence accumulated in recent years indicated that inflammatory responses can induce GADD45 expression in hematopoietic and immune cells. Bacterial endotoxin lipopolysaccharide (LPS) induces GADD45β expression *in vivo* in a range of tissues, including the liver, spleen, lung, intestine, kidney, and heart (34), as well as GADD45γ expression in the lung (62). Furthermore, GADD45β is induced by TNF-α *in vivo* and wild-type mouse embryonic fibroblasts (MEFs) (34, 63). GADD45β was also induced by IL-1 or IL-6 in the murine myelomonocytic cell line M1 (64) and M1D^+^ myeloid precursors (2, 65). Acute-phase inflammatory factors such as granulocyte-macrophage colony-stimulating factor (GM-CSF), M-CSF, G-CSF, and IL-3 were shown to induce expression of GADD45α and GADD45β in bone marrow cells (66). IL-33 and IL-12 synergistically induced GADD45β expression in CD8^+^ cytotoxic T cells (67). Like IL-33 in CD8^+^ cytotoxic T cells, IL-18 induced the expression of GADD45β and GADD45γ in CD4^+^ T helper (Th) cells, and the expression was dramatically enhanced by co-treatment with IL-12 (24). GADD45γ was also induced by cytokines IL-2 and IL-12 (3, 68, 69). However, GADD45α was not induced by IL-12, IL-18, and IL-33, but by IL-2 (10). In conclusion, inflammatory antigens and pro-inflammatory cytokines are critical in inducing GADD45 gene expression in hematopoietic and immune cells.

In addition to pro-inflammatory cytokines, TCRs have also been shown to increase the expression of GADD45. Stimulation of naïve CD4^+^ T cells with anti-CD3 and CD28 antibodies (triggering the TCR complex) resulted in upregulating the expression of GADD45β at an early time point (within 4 hours) (26). In contrast, the expression of GADD45γ in naïve CD4^+^ T cells requires prolonged stimulation with anti-CD3 and CD28 (48-96 hours) (68). This may be related to the fact that GADD45γ expression is induced by IL-2 rather than TCR signaling. Early induction of GADD45β was also observed in thymocytes *in vivo* when N15 H-2b and N15TCR transgenic mice were injected with the vesicular stomatitis virus nucleoprotein-derived octapeptide N52 ± 59 (VSV8) in the Kb major histocompatibility complex (MHC) class I molecular background (70).

### 2.3 Immunosuppressive factors

Interestingly, GADD45β expression was induced not only by immunostimulatory signals but also by immunosuppressive cytokine transforming growth factor beta (TGF-β) (71). GADD45β has been reported to be induced by TGF-β in mouse bone marrow mononuclear cell line M1, the lymphocyte line EL-4, and the mink lung epithelial cell line MvlLu (3, 72). TGF-β induces GADD45β expression in a Smad-dependent manner in pancreatic carcinoma cells (73–75). However, it is unknown whether GADD45β is required *in vivo* for the immunosuppressive function of TGF-β on immune cells.

### 2.4 Transcription factors

Nuclear factor kappa-light-chain-enhancer of activated B cells (NF-κB) is a family of inducible transcription factors that regulates multiple aspects of innate and adaptive immune functions and is a pivotal mediator of inflammatory responses (76). Several inhibitors of the NF-κB signaling pathway, including dexamethasone, cereblon E3 ligase modulator thalidomide, and proteasome inhibitor bortezomib, showed inhibitory effects on LPS-induced GADD45 expression (34). The p65 (RelA) has been reported to activate the transcriptional expression of GADD45β by binding to three κB elements on the gene’s promoter region (77). Recent studies have disclosed a novel role for the NF-κB p50 subunit in elevating GADD45α protein levels following arsenite exposure, and its mechanism is that arsenite induces the formation of IKKβ/p50 complex, which in turn inhibits GADD45α ubiquitination and leads to protein accumulation (78). Interestingly, in the cells with suppressed NF-κB gene, ROS-dependent GADD45α mRNA stabilization was observed under TNFα or arsenic stimulation (79); however, another study showed that the GADD45 mRNA expression was dramatically increased in the embryonic fibroblast cells with Ikkβ^-/-^, a kinase phosphorylates IκBα (80), these contradictory results imply that the regulation of GADD45 by NF-κB is complex.

In addition to direct regulation, NF-κB indirectly transcriptional regulates GADD45 through other transcription factors. NF-κB activation down-regulates the expression of GADD45α partially via the mediation of c-Myc (81–83). Egr-1 has also been shown to mediate between NF-κB signaling and GADD45 expression (84). The use of a Chromatin Immunoprecipitation (ChIP) assay indicated a direct interaction of Egr-1 with the promoter regions of GADD45α and GADD45β (84); a significant increase of RelA (p65)-containing NF-κB dimmers was found at κB site of Egr-1 promoter at the early stage after ultraviolet radiation b (UVB) exposure, and subsequent dramatically increased the expression of GADD45α and GADD45β in the epidermal cells (84). The transcription of GADD45α is also induced by the tumor suppressor p53 (85–87) and the Breast Cancer Gene (BRCA) (88–92).

Although the transcriptional regulation of GADD45γ is poorly understood compared to its counterparts GADD45α and GADD45β, a study showed that the GADD45γ promoter was the binding target of C/EBP family proteins (93). In addition, promoter mapping analysis identified that C/EBPβ and NF-κB/c-Rel elements were located at conserved positions of the GADD45γ promoter (93).

**Table 1** summarizes various factors involved in inducing GADD45 expression, which include environmental stimuli, pro-inflammatory and immunosuppressive factors, and transcripts.

**Table 1:** The induction of GADD45 family proteins under various stress conditions.

Stresses/Inducers		GADD45α	GADD45β	GADD45γ
Exogenous Stimulation	ionizing radiation	(17, 30–32).	–	–
	UV Radiation	(4, 8, 17, 21, 30)	(4, 17, 21, 30)	(4)
	Hypoxia	(45–47)		
	Serum Starvation	(3)	(3, 35)	(3)
	Heat Shock	–	–	(36)
	Methyl Methane sulfonate	(3, 4, 9, 17, 33, 94)	(3, 4, 17, 94)	(17, 94)
	methyl malonyl sulfonate	(30)	–	–
	Ccl4	–	(34)	–
	H2O2	(17)	(17)	–
	Anisomycin	(17)	(17)	–
	Heavy metals	(37, 38)	–	–
	Arsenic AS(III)	(39–43)	–	–
	Sodium arsenite	–	(34)	–
	Low pH	(48)	–	–
	Hyperosmotic stress	(49–52)	(50, 51)	(50)
	Cisplatin	(53–55)	–	–
	Ethanol	(56)	–	–
	Low-frequency electromagnetic fields	(57)	–	–
	peroxynitrite free radicals	(58)	–	–
	cigarette smoke condensate	(59, 60)	–	–
	Mitomycin C	(55)	–	–
Physiological Inducers	TNFα	–	(34, 63)	–
	GM-CSF/M-CSF/G-CSF/IL-3	(66)	(66)	–
	IL33 plus IL-12	–	(67)	–
	IL-18 plus IL-12	–	(24)	(24)
	Anti CD3 plus CD28	–	(26, 68, 70)	(68)
	IL-12	–		(68)
	IL-1	–	(2, 65)	
	IL-2	–	–	(3, 10, 69)
	IL-6	(3)	(2, 3, 64)	(3)
	LPS		(2, 34)	(62)
Immunosuppressive Factors	TGF-β		(3, 72–75)	
Transcription Factors	BRCA1/2	(88–91)	–	–
	P53	(85–87)	(95)	–
	NF-κB	(78, 79)	(77)	(96)
	C/EBP	(97)	–	(93)
	c-Myc	(82)	–	–
	Egr-1	(84)	(84)	–

## 3 Cellular sources and regulation

### 3.1 Myeloid Cells

Myeloid Cells are important for the innate immune system (non-specific immunity) and are immune effector cells formed during the long-term germ-line evolution of organisms. Myeloid Cells include granulocytes, monocytes, macrophages, dendritic cells (DCs), and a subgroup of leukocytes. They circulate through the blood and lymphatic system and are rapidly recruited to tissue damage and infection sites via various chemokine receptors. Within the tissues, they are activated to enhance phagocytosis, secrete various inflammatory cytokines, and play critical roles in protective immunity. Myeloid cells can also be found in tissues under steady-state conditions, where they maintain immune homeostasis and aid in tissue repair (98–100).

The GADD45 protein is essential for differentiating myeloid cells into granulocytes and macrophages. *In vitro*, bone marrow cells of GADD45α^-/-^ and GADD45β^-/-^ mice exhibited impaired myeloid differentiation and increased apoptosis under acute stimulation with various cytokines and inflammation (66). Interestingly, GADD45α^-/-^ and GADD45β^-/-^ granulocyte/macrophage progenitors regained their proliferative capacity after replanting in methylcellulose supplemented with IL-3; *in vivo*, GADD45α^-/-^ and GADD45β^-/-^ mice also displayed reduced recovery of the bone marrow myeloid after 5-fluorouracil-induced myeloablation, furthermore, GADD45α^-/-^ and GADD45β^-/-^ mice also exhibited impaired bone marrow cell responses to inflammatory stress induced by intraperitoneal administration of sodium caseinate (66). Notably, GADD45α and GADD45β deficiency led to higher proliferative capacity of immature myeloid cells. Thus, GADD45 proteins may promote the differentiation of myeloid cells and inhibit the proliferation of these terminally differentiated cells. However, GADD45γ is not required for myeloid differentiation (69).

**Figure 1:**
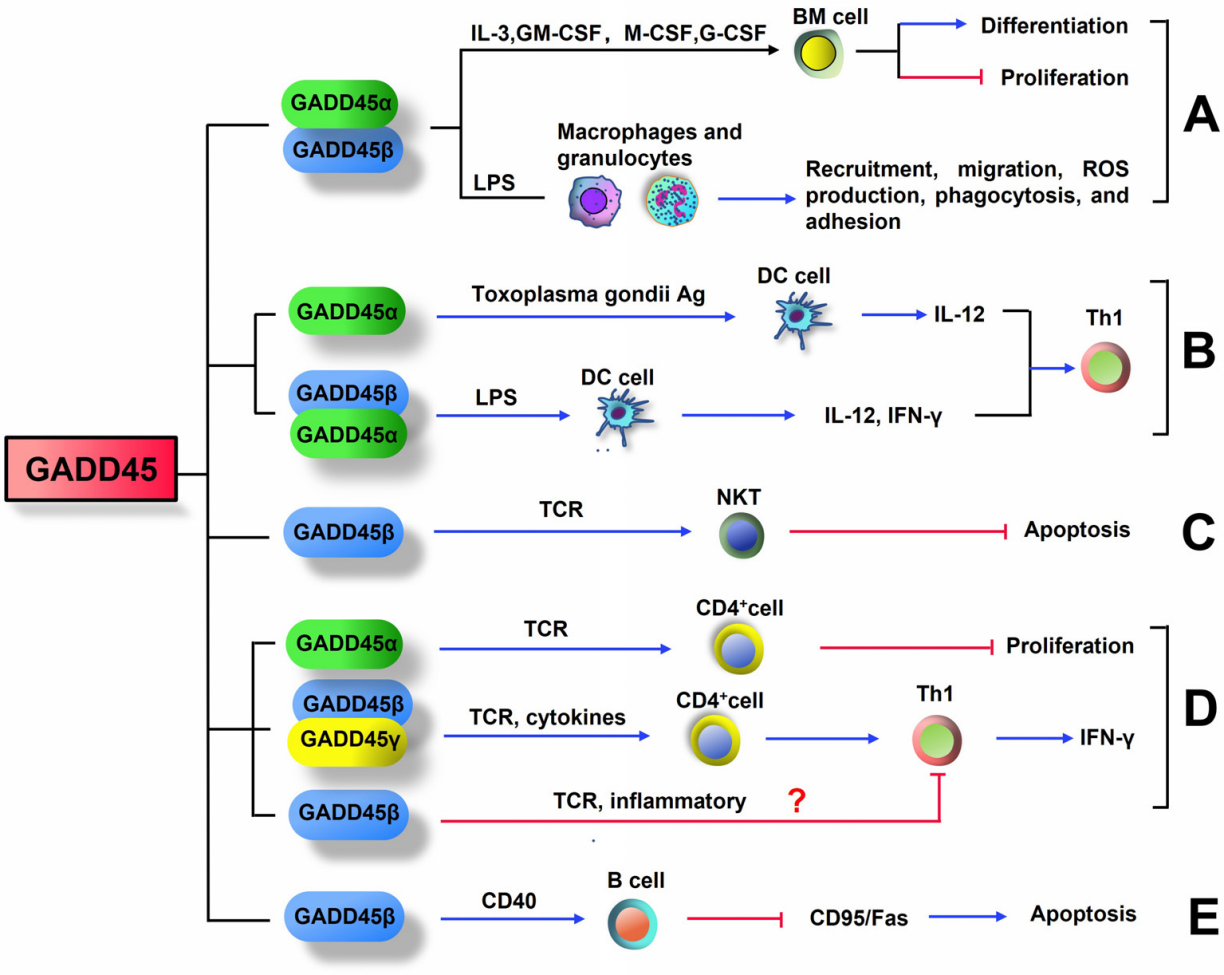
GADD45 influences the differentiation and function of immune cell. **(A)** GADD45α and GADD45β promote the differentiation of myeloid cells and inhibit the proliferation of these terminally differentiated cells. GADD45α and GADD45β promote recruitment, migration, reactive oxygen species production, phagocytosis, and adhesion of Bone marrow-derived macrophages and granulocytes. **(B)** Expression of STAg-induced GADD45α and LPS-induced GADD45β in DC cells both promotes differentiation to Th1 cells. **(C)** TCR-induced GADD45β expression in NKT cells inhibits their own apoptosis. **(D)** Stimulation of T cell receptor (TCR) increases the levels of GADD45β and GADD45γ in CD4^+^T cells, which drive inflammatory signaling for Th1 differentiation and IFN-γ expression. However, GADD45α is a negative regulator of T cell proliferation during TCR stimulation. Further studies are needed to confirm whether GADD45β can induce T cell anergy. **(E)** In B cells, GADD45β was induced by CD40, this induction inhibited Fas-mediated apoptosis.

In a mouse model of experimental sepsis, reduced recruitment of myeloid cells into the peritoneal cavity upon LPS injection was observed in GADD45α^-/-^ and GADD45β^-/-^ mice by diminishing p38 kinas and JNK activity (101). Bone marrow-derived macrophages and granulocytes from GADD45α^-/-^ or GADD45β^-/-^ mice exhibited lower migration efficiency in response to inflammatory stimuli such as LPS, N-formyl-methionine-leucine-phenylalanine, and IL-8. GADD45α and GADD45β also affect other myeloid innate immune functions, including reactive oxygen species production, phagocytosis, and adhesion (101). These data indicate that GADD45 proteins are crucial in myeloid cell differentiation, proliferation, and function (**Figure 1**).

### 3.2 Antigen-presenting cells

Antigen-presenting cells (APCs), also known as accessory cells, can ingest, process, and present antigen information to lymphocytes during the immune response. The main APCs include dendritic cells (DC), macrophages, and B lymphocytes (102, 103). Dendritic cells have the broadest range of antigen presentation and are necessary for activating naive T cells. Dendritic cells capture antigens from the environment and present them via MHC to T cells, initiating MHC-class I-restricted cytotoxic T-lymphocytes (CTL) responses and MHC-class II-restricted CD4^+^ Th responses. Dendritic cells also play a role in peripheral tolerance, which helps prevent auto-immune disease (104, 105). Bone marrow-derived dendritic cells from GADD45α-deficient mice exhibited less activation of the classical MKK3/6-p38 mitogen-activated protein kinase (MAPK) cascade, lowered level Th1 cytokine IL-12 and IFN-γ production, as well as decreased expression of the co-stimulatory molecule CD40 upon stimulation with soluble antigens from toxoplasma gondii (STAg) (106). In addition, GADD45β-deficient dendritic cells produced less IFN-γ and IL-12 upon stimulation with LPS (26). Therefore, the activation of canonical MAPK signaling by GADD45 proteins is crucial for generating a Th1 response via the activation of dendritic cells (**Figure 1**).

### 3.3 Natural killer T cells

Natural killer T (NKT) cells are a unique subset of lymphocytes that link the innate and adaptive immune system, possessing characteristics of NK cells and memory T cells. They constitute approximately 1% of all peripheral blood T cells (107, 108). Unlike most conventional T cells, NKT cells do not recognize peptide antigens bound to MHC class I or MHC class II molecules. Instead, these cells directly recognize glycolipids (such as α-galactosylceramide), including exogenous and endogenous lipid antigens presented by MHC-like CD1d molecules in antigen-presenting cells (109). Upon activation, NKT cells can produce many cytokines and chemokines that play an immunoregulatory role in autoimmune diseases and antimicrobial immunity (110, 111). Interestingly, compared with conventional T cells, NKT cells are more resistant to TCR-induced apoptosis, mainly due to the preferential expression of anti‐apoptotic genes, such as GADD45β (112) (**Figure 1**). However, so far, there are no reports on how GADD45β regulates the survival of NKT cells. Thus, the importance of the GADD45 protein in NKT cell biology requires further investigation.

### 3.4 T cells

For adaptive immunity, most of the work on GADD45 proteins has concentrated on T cells. GADD45β is vital for Th1 responses; in CD4^+^ T cells, GADD45β expression rapidly increased following T cell receptor (TCR) activation and inflammatory stimulation (24, 26). T cells transfected with GADD45β-retrovirus promote IFN-γ secretion after IL-12 and IL-18 stimulation, thereby driving Th1 differentiation (24). GADD45β-deficient CD4^+^ T cells showed impaired responses to TCR signal or inflammatory cytokines stimulation, suppressed the activation of extracellular regulated protein kinases (ERK), p38, and JNK activity, and reduced cytokine production (26). These effects can be compensated by GADD45 proteins (17) and enhanced by a dominant-negative version of MEKK4 (24). In addition, GADD45β, GADD45γ, and MEKK4 comprise a pathway that enhances IFN-γ production and Th1-mediated immunity responses (113). On the contrary, another study reported that GADD45β deficient Th1 cells increased the proliferation of the cells in response to TCR or inflammatory signals (28). Thus, GADD45β and GADD45γ serve as molecular “double-edged swords” and play a key role in Th1-type immune response; this role is important for producing Th1 cells during the initiation phase of the immune response; however, it is also used in the later phase to shut down the immune response. The absence of such a regulatory mechanism would seriously affect the initiation and termination of the immune response.

GADD45γ was also strongly induced during T cell activation, and the expression level is higher in Th1 cells than in TH2 cells (68). Under TCR-stimulation conditions, GADD45γ^-/-^ Th1 cells exhibit reduced p38 and JNK MAPK activity, less IFNγ production, and deficient activation-induced cell death (AICD) (68). Moreover, the lack of GADD45γ in mice reduced contact hypersensitivity of Th1 cells, indicating that the cell responses were also impaired *in vivo* (68). Therefore, GADD45γ mediates the function of Th1 cells by activating the p38 and JNK pathways (**Figure 1**).

In contrast to GADD45β/γ, GADD45α is a negative regulator of T-cell proliferation (**Figure 1**). Compared to wild-type cells, GADD45α^-/-^ T cells have a lower activation threshold and proliferate to a greater extent following primary T cell receptor activation (114). Another study showed that resting T cells from GADD45α^-/-^ mice had spontaneously increased p38 activity without MAPK kinase activation, and the p38 activity was explicitly inhibited *in vitro* by recombinant GADD45α (115).

T cell anergy is a tolerance mechanism in which the lymphocyte is intrinsically functionally inactivated after encountering an antigen but remains alive for prolonged periods in a hypo-responsive state (116, 117). T cell anergy can be mediated by the nuclear factor of activated T cells (NFAT) as well as early growth response 2 (Egr2) and Egr3 (118). GADD45β was identified as a gene induced during T cell anergy by DNA microarray analysis (119). Deltex1 (DTX1) was a transcription target of the NFAT that participated in T cell anergy (120). Importantly, DTX1 also regulated the expression of GADD45β. However, further studies are needed to demonstrate the role of GADD45β in T cell anergy.

### 3.5 B cells

B cells, also known as B lymphocytes, are a type of white blood cell of the lymphocyte subtype, which function in the humoral immunity component of the adaptive immune system (121, 122). It has been reported that in B cells, GADD45β was induced by CD40, a TNF receptor superfamily member providing costimulatory signals to B cells. And this induction inhibited CD95/Fas-mediated (i.e., extrinsic) apoptosis. In addition, GADD45β impaired the Fas-induced apoptotic cascade at mitochondria but did not impede the ‘intrinsic’ pathway of apoptosis (123). These results suggest that GADD45 is an anti-apoptotic protein in B cells, which can protect B cells from AICD (**Figure 1**). However, the exact mechanism of the effect of GADD45β on apoptosis is still unclear.

## 4 The main regulative mechanism of GADD45

### 4.1 P38 mitogen‐activated protein kinase pathway

MAPK cascade is a crucial immune-responsive signaling pathway in eukaryotic cells. They are located downstream of membrane sensors/receptors and coordinate with cellular responses to convert extracellular stimuli (antigens/pathogens) into intracellular responses, which enhances the body’s immunity and ability to resist infections, thus enabling the body to adapt and survive in an ever‐changing environment (124). The MAPK family includes the extracellular signal-regulated kinases ERK1, ERK2, and ERK5, the c-jun NH2-terminal kinases JNK 1, JNK 2, and JNK 3, the four p38 enzymes, p38α, p38β, p38γ, and p38δ, and big MAP kinase 1 (125). p38 MAPKs are described as stress-activated protein kinases (SAPKs) because they are frequently activated by a wide range of environmental stresses and cytokines to induce inflammation. Thus, they play a critical role in the host defense system (126).

**Figure 2:**
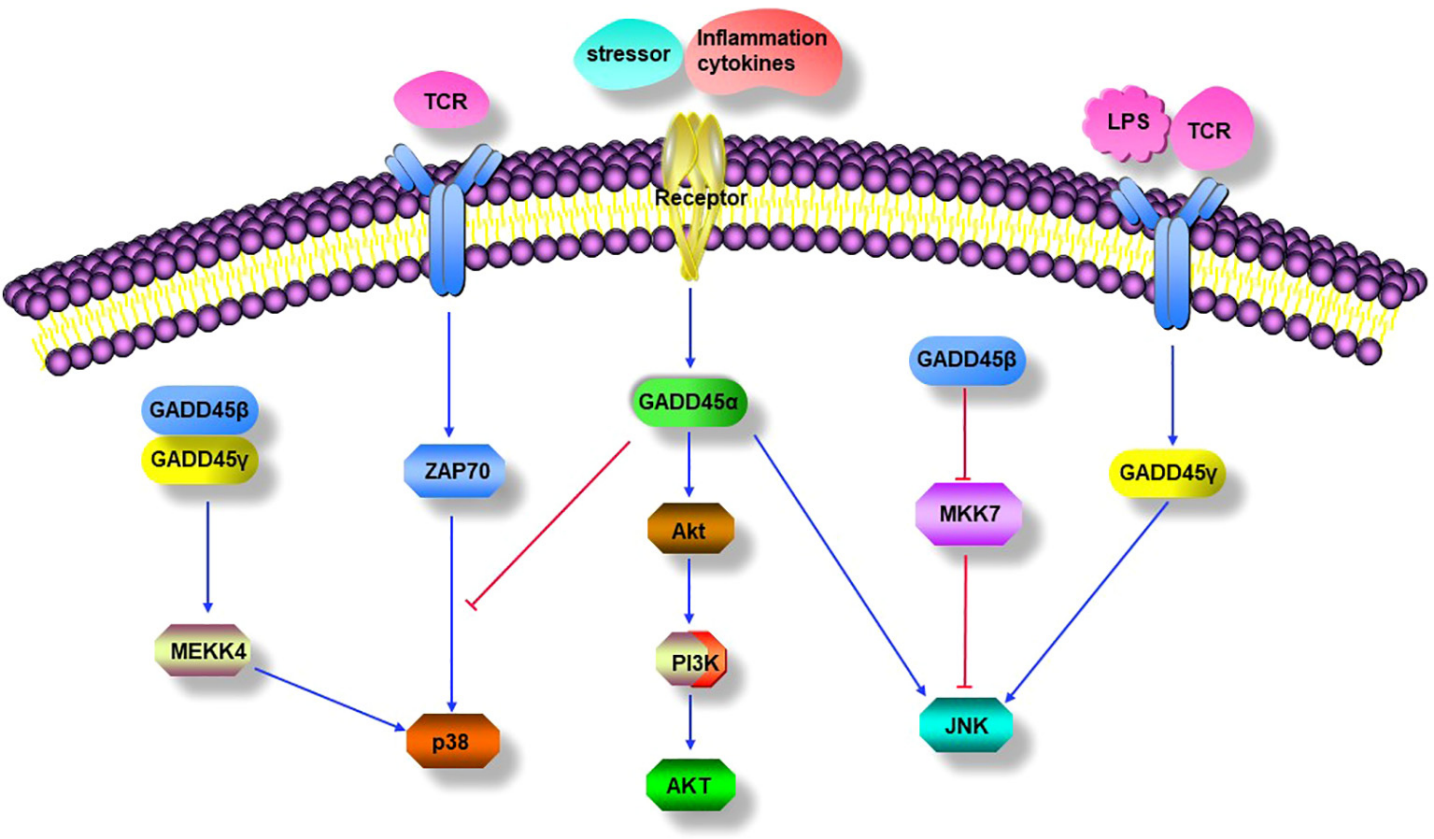
GADD45 modulated signaling pathways. GADD45 proteins mediate activation of the classical p38 MAPK pathways. GADD45α inhibits the TCR-mediated alternative p38 activation pathway. Stressor or inflammation cytokines induced GADD45α positively modulated PI3K/Akt signaling pathway. GADD45α and GADD45γ promote the JNK MAPK signal pathway, while GADD45β negatively modulates the activation of the JNK signal pathway by downregulating the activity of MKK7.

An increasing number of studies have shown that all of the GADD45 proteins can activate the p38 MAPK pathway in T cells, thereby affecting the production of IFN-γ and other pro-inflammatory-related mediators (24, 26, 68, 113, 127–129) (**Figure 2**). Compared with CD4^+^ T cells from MEKK4^+/+^ mice, CD4^+^ T cells from MEKK4^-/-^ mice showed a decrease in p38 activity and IFN-γ production after TCR or IL-12 and IL-18 stimulation (113). Overexpression of GADD45β or GADD45γ promotes IFN-γ secretion in MEKK4^+/+^ T cells but not in MEKK4^-/-^ cells or cells treated with a p38 inhibitor (113). Thus, GADD45β and GADD45γ increase p38 activity by regulating MEKK4, which leads to increased IFN-γ production (**Figure 2**). In contrast, Yang, J et al. reported that GADD45β binds to MEKK4 and activates the p38 MAPK pathway in CD4^+^ T cells, which was required for cytokine-induced IFN-γ transcription but not for TCR-induced IFN-γ transcription; inhibition of the p38 MAPK pathway selectively inhibited cytokine-induced IFN-γ production, but not TCR-induced IFN-γ production, further confirming this point (24).

As mentioned above, GADD45β and GADD45γ activated p38 MAPK through the classical kinase cascade, which is crucial for T-cell differentiation into Th1 cells. However, GADD45α has distinct roles in regulating p38 MAPK activity in T cells. TCR signaling can activate p38 through an alternative pathway unrelated to the classical MAPK cascade (**Figure 2**). In the alternative pathway, TCR activates the tyrosine kinase ZAP70, which phosphorylates p38 on Tyr323 and subsequently auto-phosphorylates its residues Thr180 and Tyr182, leading a full activation of p38 (130). Genetic replacement of Y323F impaired full activation of p38 and IFN-γ synthesis in Th1 cells, suggesting that the alternative pathway is required for proinflammatory Th cell functions (131). Furthermore, the alternative p38 pathway up-regulated the transcription factors NFATc1 and interferon regulatory factor 4 (IRF4) at the molecular level, which was required for proliferation and cytokine production in T cells (132, 133). Interestingly, GADD45α has been reported to have an inhibitory effect on the alternative p38 activation pathway in T cells, as evidenced by the spontaneous phosphorylation of 38 Tyr323 in GADD45α^-/-^ mouse T cells in the absence of MAPKK activity; the mechanism by which GADD45α restrains p38 activity is by blocking its Tyr323 phosphorylation and directly inhibiting Tyr323-phosphorylated p38 activity, and further study showed that the inhibition of p38 Tyr323 phosphorylation by GADD45α was through suppression of Zap70 rather than MKK6 (115). The results indicate that GADD45α may restrain T cell p38 activation by regulating the TCR signaling pathway (**Figure 2**). However, the opposite effects of the GADD45 family proteins on the activity of p38 were found between GADD45β/GADD45γ and GADD45α, unlike the inhibitory effect of GADD45α on p38, GADD45β/GADD45γ can significantly enhance the kinase’s activity, indicating the complexity of immune regulation by GADD45 family proteins in T cells (28, 115).

### 4.2 c-Jun N-terminal kinases mitogen‐activated protein kinase pathway

Like p38 MAPKs, JNK MAPKs can be activated by environmental and genotoxic stresses. They have critical roles in inflammation and tissue homeostasis, as they control cell proliferation, differentiation, survival, and the migration of specific cell types (134, 135).

Induction of GADD45α in placental explanted by stressors or inflammatory cytokines can activate the JNK MAPK pathway (127). GADD45γ^-/-^ mice lacked AICD and lower contacted hypersensitivity, and Th1 cells from GADD45γ^-/-^ mice have significantly diminished ability to activate JNK MAPK in response to TCR signaling and dramatically reduce the production of IFN-γ; these effects were consistent with impairment of the JNK MAPK pathway (68). When GADD45γ was blocked in Th1 cells, LPS failed to activate JNK and, therefore, is unable to upregulate the expression of pro-inflammatory cytokines, whereas, in GADD45γ over-expressing Th1 cells, LPS enhanced JNK activation and increased the production of pro-inflammatory cytokines (136). In addition, the JNK inhibitor had a more inhibitory effect on LPS-induced TNFα production in GADD45γ over-expressing cells than in GADD45γ knocked-down cells, suggesting that GADD45γ may act upstream of JNK to mediate TNFα synthesis (136). In contrast, GADD45β had an opposite effect on JNK, and forced expression of GADD45β in human fibroblast-like synoviocyte (FLS) blocks TNF-induced MKK7 activation, implying that GADD45β attenuates JNK pathway signaling. Moreover, in a KB/xN serum-induced arthritis model, GADD45β^-/-^ mice exhibited a significant increase in JNK phosphorylation and a worsening of arthritic symptoms (137). These data suggest that GADD45α and GADD45γ promote the JNK-MAPK signaling pathway, while GADD45β inhibits JNK-MAPK activity by impairing MKK7 activity (**Figure 2**).

### 4.3 PI3K/AKT1 pathway

The PI3K/Akt pathway is an intracellular signaling transduction pathway that promotes metabolism, proliferation, cell survival, growth, and angiogenesis in response to extracellular signals (138–141). The regulatory mechanisms and biological functions of the PI3K/Akt signaling pathway are essential in many human diseases, including ischemic brain injury, neurodegenerative diseases, tumors, and inflammatory diseases (142–146).

In a mouse model of acute lung injury, GADD45α^-/-^ mouse showed severe dysregulation of B-cell receptor signaling compared to wild-type mice; Western blot analysis of lung homogenates confirmed a ∼50% reduction in Akt protein levels in GADD45α^-/-^ mice, accompanied by a marked increase in Akt ubiquitination, suggesting that GADD45α is involved in PI3K/Akt signaling regulation. Electrical resistance measurements across human lung endothelial cell monolayers with either reduced GADD45α or Akt expression (siRNAs) revealed a significant enhancement of LPS-induced human lung endothelial barrier dysfunction that was attenuated by overexpression of a constitutively active Akt1 transgene (146). In murine models of radiation- and bleomycin-induced lung injury, GADD45α^-/-^ mice had decreased levels of total and phosphorylated Akt in the lung compared to wild-type mice, whereas increased Radiation-Induced Lung Injury (RILI)susceptibility was observed in both Akt^+/-^ mice and mice treated with an Akt inhibitor from 1 week before to irradiation. Furthermore, overexpression of a constitutively active Akt1 transgene reversed RILI-susceptibility in GADD45α^-/-^ mice (147). Thus, it suggests that GADD45α may be located upstream of the PI3K/Akt signaling pathway and positively modulate this signaling pathway (**Figure 2**).

## 5 GADD45 and autoimmune disease

GADD45 is induced by different stimuli and expressed in different cells, exhibiting distinct biological functions and effects in various inflammatory and autoimmune diseases.

### 5.1 Rheumatoid arthritis

**Figure 3:**
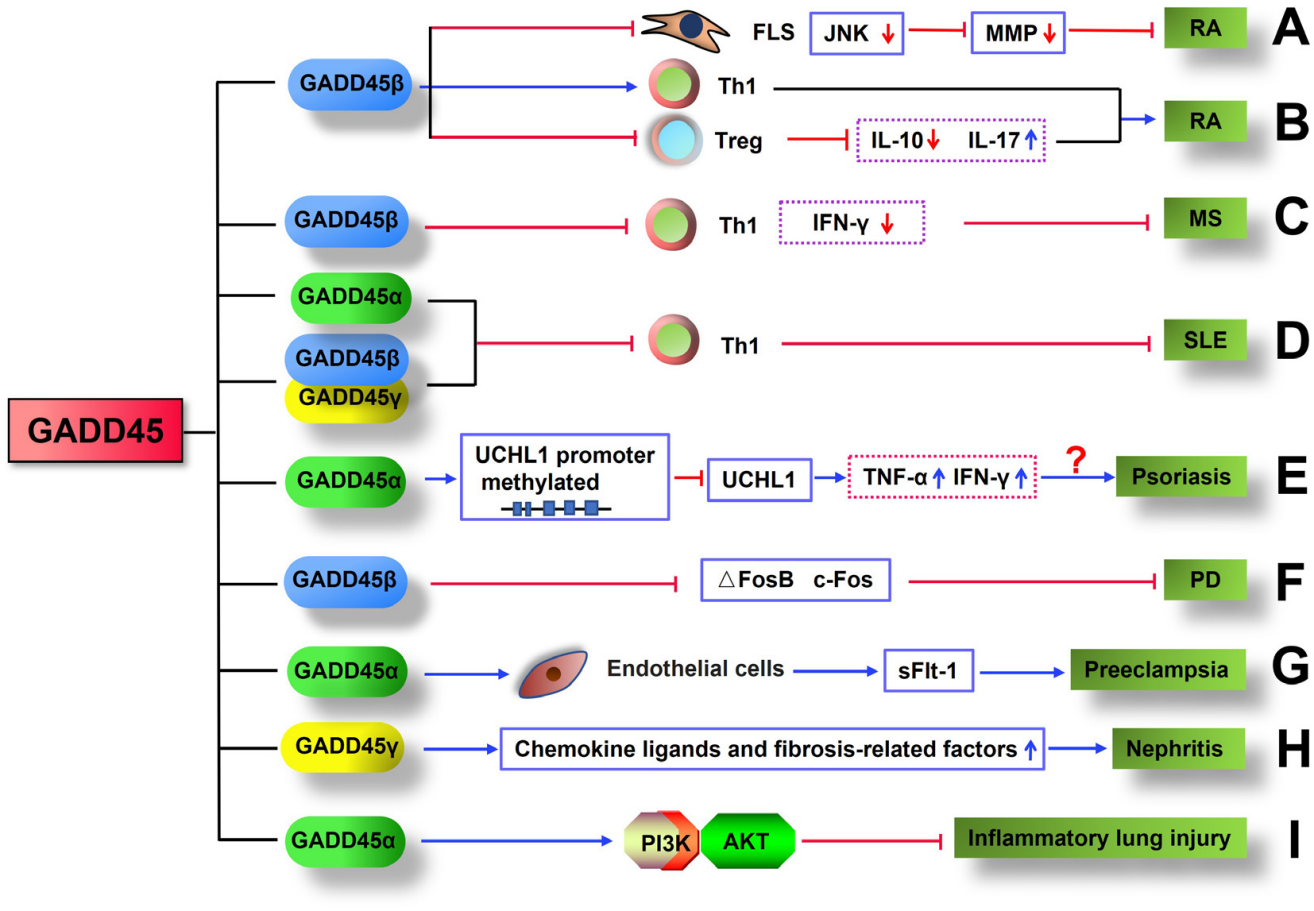
GADD45 is involved in the pathology of inflammatory disease. **(A)** GADD45β inhibited K/BxN serum-induced arthritis by impairing TNF-induced JNK signaling activation and reducing MMP expression. **(B)** GADD45β exacerbated CIA by increasing Th1 cell infiltration in joints, reducing the number of Treg cells, decreasing IL-10 expression, and elevating IL-17 production. **(C)** GADD45β inhibits MS by limiting the proliferation of Th1 cells and the production of IFN-γ. **(D)** GADD45 protein limited the development of SLE by inhibiting the proliferation of Th1 cells. **(E)** GADD45α may promote the occurrence of psoriasis by inhibiting UCHL1 expression through upregulation of UCHL1 methylation, which in turn promotes the production of inflammatory factors. **(F)** GADD45β suppresses PD by downregulating the expression of ΔFosB and c-Fos. **(G)** GADD45α contributes to the development of preeclampsia with upregulation of sFlt-1 secretion in endothelial cells. **(H)** GADD45γ exacerbates the progression of nephritis by increasing the expression of chemokine ligands and fibrosis-related factors. **(I)** GADD45α restrains inflammatory lung injury by activating the PI3K/AKT.

Rheumatoid arthritis (RA) is one of the most common chronic autoimmune diseases characterized by progressive articular damage, functional loss, and comorbidity (148). Recently, studies showed that GADD45 may play an attenuated or aggravated role in autoimmune diseases such as RA. It was found that the levels of GADD45β mRNA and protein in RA patients were significantly lower than in healthy controls (29), especially in synovial fibroblasts of RA patients (137). Overexpression of GADD45β in human FLS impaired TNF-induced JNK signaling activation, activator protein 1 (AP-1) activity, and reduced MMP expression (137). The above results were corroborated by the fact that joints of GADD45β^-/-^ mice in K/BxN serum-induced arthritis exhibited a dramatic increase in JNK activity, upregulation of matrix metalloproteinases 3 and 13, aggravation of joint inflammation, and higher clinical scores (137) (**Figure 3**). Du Fang et al. found that compared with healthy controls, Th1 cells in the synovial fluid (SF) of RA patients had higher levels of GADD45β and lower apoptotic rate; more importantly, GADD45β RNAi can reverse the resistance of Th1 cells to apoptosis, confirming the anti-apoptotic effect of GADD45β in Th1 cells (149) (**Figure 3**). Furthermore, GADD45β deficiency mice in collagen-induced arthritis (CIA) showed significantly lower arthritis severity and joint destruction, elevated IL-10 expression, decreased IL-17 production, and increased Treg cells compared with WT mice (150) (**Figure 3**). However, K/BxN serum-induced arthritis and experimental autoimmune encephalomyelitis (EAE) were alleviated by GADD45β, suggesting that GADD45β plays a complex role in regulating adaptive immunity and can enhance or suppress inflammation according to different disease models.

### 5.2 Multiple sclerosis

Multiple sclerosis (MS) is a common immune-mediated disorder affecting the central nervous system (151). While the cause is unclear, the underlying mechanism is thought to be either destruction by the immune system or failure of the myelin-producing cells (152). EAE is a murine model of human MS, mainly caused by the infiltration of autoimmune Th1 cells into neuronal tissues such as the brain and spinal cord. GADD45β (28) and GADD45γ (28, 68) were shown to inhibit the proliferation and activation of Th1 cells in response to TCR signaling *in vitro*. More importantly, in GADD45β-deficient mice, CD4^+^ T cells rapidly proliferated and infiltrated the nervous system in EAE induced by myelin oligodendrocyte glycoprotein (MOG) peptide. Compared with wild-type mice, mice lacking GADD45β exhibited more aggravated and prolonged clinical EAE signs and symptoms in response to myelin immunization; mice with double deficiency of GADD45β and GADD45γ spontaneously developed Systemic lupus erythematosus (SLE) and autoimmune lymphoproliferative syndrome (ALS); the EAE symptoms became even more pronounced when GADD45β deficient naïve or CD4^+^ T cells were transferred into immunodeficient (Rag1^-/-^) mice; at the late time points, the mice exhibited more severe signs of inflammation, such as high levels of IFN-γ in CD4^+^ Th cells, marked leukocyte infiltration, and activation of microglia cells (28) (**Figure 3**). In addition, compared with GADD45^+/+^ Th1 cells, GADD45^-/-^ Th1 cells showed more vital proliferation ability and were more resistant to the induction of apoptosis (28). Thus, GADD45β and GADD45γ are required for AICD and inhibiting proliferation and activation of Th1 cells in response to TCRs and cytokines stimulation in EAE (28, 68). These findings suggest that regulation of T cells by GADD45β and GADD45γ are critical for maintaining autoimmune homeostasis in the diseases.

### 5.3 Systemic lupus erythematosus

Systemic lupus erythematosus (SLE) is an autoimmune disease in which the immune system mistakenly attacks healthy cells and tissues throughout the body (153). As mentioned earlier, GADD45α negatively regulated the proliferation of CD4^+^T cells. Importantly, GADD45α^-/-^ mice spontaneously developed an autoimmune disease similar to human SLE, characterized by high titers of anti-dsDNA, anti-ssDNA, and anti-histone autoantibodies. At nine months of age, GADD45α^-/-^ mice exhibited signs of severe autoimmune glomerulonephritis and hematological disorders accompanied by reduced numbers of leukocytes and lymphocytes in peripheral blood (114) (**Figure 3**). Mice with a combined GADD45β and GADD45γ deficiency also spontaneously developed SLE (28) (**Figure 3**). Furthermore, two single nucleotide polymorphisms (SNPs) of GADD45 have been identified as associated with autoimmune diseases, namely, the GADD45α 589GG+GC is linked with rheumatoid factor (RF), and the GADD45β -712CT genotypes are related to anti-RNP antibodies in SLE patients (29). Thus, GADD45 gene members might play negative regulatory roles in the pathogenesis of SLE.

### 5.4 Psoriasis

Psoriasis is a chronic, long-lasting, noncontagious autoimmune disease characterized by raised areas of skin with chronic, symmetrical, erythematous, scaling papules and plaque (154, 155). GADD45α was upregulated in peripheral CD4^+^ T cells of psoriasis patients, especially the infiltrating T cells in the dermis of damaged skin, but the level of GADD45α was lower in the epidermal cells; GADD45β also exhibited a similar expression pattern to GADD45α in the patients with psoriasis; in addition, the expression of GADD45α positively correlated with IFN-γ and TNF-α in the affected skin of psoriasis patients, a positive correlation was also observed between GADD45β and TNF-α (156). Thus, increased expression of GADD45α and GADD45β in psoriatic leukocytes may be related to the pro-inflammatory environment in the skin (114, 137, 156) (**Figure 3**). DNA demethylation is a process in which a methyl group is removed from DNA; it generally results in the activation of gene expression by altering the interaction of the cell’s transcription machinery with DNA. GADD45α has been shown to participate in DNA demethylation of the promoter of Ubiquitin C-terminal hydrolase L1(UCHL1); as a deubiquitinase, UCHL1 is involved in the controls keratinocyte proliferation and inflammation in psoriasis; hypermethylated UCHL1 promoter was found in the psoriatic lesioned skin and associated with a lower level of GADD45α protein, indicating that the demethylation of UCHL1promoter by GADD45α increases the expression of UCHL1 protein in psoriatic damaged skin (156). Moreover, the silencing of GADD45α in skin squamous cells increased inflammatory cytokines such as IL-1, IL-6, and TNF α (157) (**Figure 3**). Thus, GADD45α downregulates immune response and inhibits keratinocyte proliferation by increasing UCHL1 demethylation, thereby controlling the progression of psoriasis.

### 5.5 Parkinson’s disease

Parkinson’s disease (PD) is a progressive neurodegenerative disease that affects peripheral organs as well as the central nervous system, and neuroinflammation plays a critical role in its pathological process. Growing evidence suggests that both innate and adaptive immune systems are involved in the pathogenesis of PD (158–161). Previous studies showed that in a 6-hydroxydopamine (6-OHDA) induced Parkinson’s mouse model, GADD45β expression was lower in the dorsal striatum (162). Interestingly, after administration of dopamine precursor 3,4-dihydroxyphenyl-L-alanine (L-DOPA), the expression of GADD45β in the dorsal striatum of 6-OHDA-induced PD mice was dramatically higher than that of the control group mice; the level of GADD45β was positively correlated with the dose of L-DOPA. More importantly, compared with wild-type mice, mice lacking GADD45β exhibited more persistent abnormal involuntary movements (AIMs) after repeated administration of L-DOPA. In contrast, injecting AAV-GADD45β into the dorsal striatum of GADD45β^-/-^ mice significantly decreased AIM scores. In the diseased striatum, compared to GADD45β^+/+^ mice, mice lacking GADD45β had significantly increased expression of ΔFosB (a transcription factor that is a critical mediator in maladaptive neuroplasticity in PD) and c-Fos (immediate early gene, a mark of acute neuronal activity) (162, 163) (**Figure 3**). These data indicate that the increased expression of GADD45β induced by repeated administration of L-DOPA may be beneficial in reducing the symptoms of PD.

### 5.6 Preeclampsia

Preeclampsia is a disorder of pregnancy characterized by the onset of high blood pressure and often with a large amount of protein in the urine (164). Excessive and progressive activation of the immune system, along with an increase in proinflammatory cytokines and antiangiogenic factors in the fetal placental units and maternal vascular endothelium, are associated with the pathogenesis of preeclampsia (165–167). Compared with pregnant women with non-preeclampsia, patients with preeclampsia have elevated levels of GADD45α mRNA and protein in placental tissue (128). In addition, endothelial cells and trophoblast cells in patients with preeclampsia exhibited a high level of p38 protein, which is a downstream effector of GADD45α; furthermore, GADD45α and sFlt-1 (a circulating factor that plays a key role in the pathophysiological-related symptoms of preeclampsia) were found to be co-expressed in preeclamptic placental endothelial cells (128) (**Figure 3**). *In vitro*, placental explant culture showed that hypoxia, angiotensin II, and inflammatory cytokines can induce the expression of GADD45α, which activated p38 and JNK and increased sFlt-1 secretion (127). RNAi-mediated knockdown of GADD45α abolished p38 activity and significantly reduced sFlt-1 levels in placental explant culture medium (127, 128). These observations indicate that GADD45α signaling may serve as a hub linking placental stresses and the pathogenesis of preeclampsia. However, Yonghui Yu et al. found that knocking out GADD45α in mouse embryonic fibroblasts (MEFs) increased the activity of the JNK/p38 pathway, and overexpression of HA-GADD45α in GADD45α^-/-^ MEFs reduced the pathway activity (168). The dual effect of GADD45α on the JNK/p38 pathway may be due to different cells and diseases; further studies are needed to elucidate this phenomenon.

### 5.7 Nephritis

Nephritis is inflammation of the kidneys, which may involve the glomeruli, tubules, or interstitial tissue surrounding the glomeruli and tubules. GADD45γ expression is increased in rat kidneys with ureteral obstruction and renal biopsy tissue obtained from patients with chronic glomerulonephritis (129). Adenovirus-mediated expression of GADD45γ in cultured renal tubular cells activated p38 and significantly upregulates chemokine ligands and fibrosis-related factors; silencing the expression of GADD45γ significantly blunted the inflammatory and fibrotic mediators and monocyte infiltration in the kidneys of rats with ureteral obstruction (129) (**Figure 3**). Compared with patients with negative GADD45γ mRNA in urine, patients with positive GADD45γ mRNA in urine had 3-4 fold faster deterioration of renal function and significantly reduced renal survival rate (169). Furthermore, GADD45γ promoted apoptosis of glomerular mesangial cells (170, 171) and renal tubular cells (172, 173). These results suggest that GADD45γ may enhance the production of factors promoting the pathogenesis of kidney disease, which suggests that this protein may have the potential to become a new therapeutic target for nephritic disease.

### 5.8 Inflammatory lung injury

Inflammatory lung injury is a common and severe morbid inflammatory syndrome characterized by the onset of extensive lung inflammation, which can be induced by pathogenic microbial infection, trauma, pneumonia, and drugs (174). GADD45α expression was increased in ventilator-induced lung injury (VILI) models (175). In lipopolysaccharide (LPS)-, ventilator- and radiation-induced lung injury models, total cells, protein, albumin, and cytokines in bronchoalveolar lavage fluid were significantly higher in GADD45α^-/-^ mice than in wild-type mice, indicating that GADD45α plays a crucial role in reducing lung injury (146, 147). Furthermore, after two weeks of treatment with bleomycin (0.25 U/kg IT), the pulmonary fibrosis in GADD45α^-/-^ mice was significantly higher than in wild-type mice (147). Compared with wild-type mice, GADD45α^-/-^ mouse lungs showed reduced considerably total Akt protein and its phosphorylation levels and exhibited more severe radiation-induced lung injury (RILI), whereas overexpression of Akt1 attenuated RILI (146, 147). These findings suggest that GADD45α may reduce susceptibility to acute lung injury factors by upregulating the PI3K/AKT signaling pathway (**Figure 3**). Thus, it may have the possibility to serve as a new therapeutic target for inflammatory lung injury in a clinical setting.

### 5.9 Graves’ disease

Graves’ disease (GD) is a thyroid-specific autoimmune disorder primarily due to reduced tolerance to thyrotropin receptors (176, 177). It is the most common cause of hyperthyroidism (178). The mRNA levels of Gadd45α and β were elevated in patients with active Graves’ disease compared to normal controls. The mRNA levels of these two GADD45 isoforms were even higher in Graves’ disease patients with normal thyroid function than in controls (179). These results suggest that GADD45 is involved in regulating Graves’ disease, but its effects on GADD45 and the exact regulatory mechanism of the disease require further study.

## 6 Conclusion

The GADD45 family genes are widely expressed in body tissue and cells and play important roles in various autoimmune diseases. The GADD45α is increased in preeclampsia and VILI, aggravating preeclampsia but attenuating acute lung injury. The levels of GADD45α and GADD45β are lower in psoriatic lesion skin but higher in Grave’s disease, suggesting they may be involved in regulating the pathogenesis of these two diseases. GADD45α^-/-^ mice spontaneously developed an autoimmune disease similar to human SLE. Mice with a combined GADD45β and GADD45γ deficiency also spontaneously developed SLE, indicating a potential inhibiting role for GADD45 in SLE. Mice deficient in GADD45β show more severe and prolonged clinical signs and symptoms of EAE in response to myelin immunoreactivity. The levels of GADD45β in RA patients’ synovial tissues and synovial fibroblasts were significantly reduced. The regulation of RA by GADD45β is somewhat complex. The research showed that GADD45β attenuated K/BxN serum-induced arthritis but exacerbated CIA-induced arthritis. GADD45β has also been implicated in the pathogenesis of Parkinson’s disease. GADD45γ has been shown to be related to GADD45γ nephritis, in which abnormally expressed GADD45γ protein leads to end-stage kidney disease and links to IgA nephropathy and mesangioproliferative glomerulonephritis. The accumulated data indicate that the GADD45 family protein deeply participates in autoimmune disease regulation and may have the potential to act as a therapeutic target and diagnostic marker for a number of autoimmune diseases.

Each of the GADD45 family proteins possesses distinct expression patterns under various stress conditions (**Table 1**). They target the same and/or different signaling pathways (**Figure 2**), thus resulting in they have overlapping but unique functions in autoimmune diseases (**Figures 1**, **3**). A growing body of *in vitro* and *in vivo* data has provided a solid foundation to support the regulatory role of GADD45 in autoimmune diseases. However, there are still some scientific questions that need to be addressed. GADD45β has been observed to have opposing effects on K/BxN serum- and CIA-induced arthritis in mice. Why does the same isoform of GADD45β have different roles in the same autoimmune disease? Obviously, further studies are required to elucidate the exact molecular mechanism behind this effect. Studies have shown that the effects of different GADD45 family proteins in different autoimmune diseases are different. Obviously, it is necessary to clarify the roles of different GADD45 family subtypes in various diseases and even the same disease and reveal their immunoregulatory network of GADD45 isoforms in diseases. In light of this, future research effects should focus on analyzing signaling pathways regulated by each isoform of the GADD45 family in different diseases, thereby establishing the relationship between gene subtypes and diseases. This will help to provide a precise prevention and treatment strategy for autoimmune diseases caused by GADD45 abnormalities and help researchers identify new therapeutic targets and biomarkers.

## Glossary

Ads: Autoimmune diseases
GADD45: Growth arrest and DNA damage-inducible 45
APCs: antigen present cells
ML-1: myeloid leukemia cell line
PKO: colon adenocarcinoma cell line
BM: Bone marrow
CHO: Chinese hamster ovary
MMS: methyl methane sulfonate
LPS: lipopolysaccharide
GM-CSF: granulocyte–macrophage colony-stimulating factor
TGF-β: transforming growth factor beta
EL-4: mouse T-cell lymphoma cell line
CCL64: Mink cell line Mv 1 Lu
DCs: dendritic cells
STAg: Toxoplasma gondii
MHC: major histocompatibility complex
CD28: cluster of differentiation 28
flCTLA4: full-length Cytotoxic T-lymphocyte antigen 4 mRNA
sCTLA4: soluble Cytotoxic T-lymphocyte antigen 4
liCTLA4: ligand-independent Cytotoxic T-lymphocyte antigen 4
NF-AT: nuclear factor of activated T cells
cAMP: Cyclic adenosine monophosphate
TGN: trans-Golgi network
GTPases: guanosine triphosphatases
ARF-1: adenosine diphosphate ribosylation factor-1
PLD: phospholipase D
CAP-1: clathrin adaptor protein-1
CAP-2: clathrin adaptor protein-2
IL-2: interleukin 2
TCR: T cell antigen receptor
Bcl-xL: apoptosis regulator Bcl-X
LAT: linker for activation of T cells
NF-κB: transcription factors nuclear factor B
Treg: regulatory T cell
DCs: dendritic cells
GRB2: growth factor receptor bound protein
SOS: Son-of-Sevenless
SYP: tyrosine phosphatase synaptophysin
CXCR4: C-X-C chemokine receptor type 4
PIP3: Phosphatidylinositol (3,4,5)-trisphosphates
PH: pleckstrin homology
PDK1: PH domain kinase 1
mTORC2: rapamycin complex 2
PP2A: serine/threonine phosphatase PP2A
SHP2: tyrosine phosphatase SHP2
BAD: BL2 associated agonist of cell death
cdCTLA: 4 cytoplasmic domain of CTLA4
Tfr: follicular regulatory T
Tfh: follicular helper T
GC: germinal center
RA: Rheumatoid Arthritis
RF: rheumatic factor
ACPA: anti-citrullinated protein antibodies Treg, regulatory T cell
Tcon: conventional T
IDO: indoleamine 2,3-dioxygenase
SLE: Lupus Erythematosus
MS: Multiple sclerosis
CNS: central nervous system
IFN-γ: interferon-γ
AP: cell-Penetrating Peptide (AP)-conjugated
ctCTLA4: CTLA4, cytoplasmic domain
T1D: type1 Diabetes
NOD: non-obese diabetes
CRP: C-reactive protein
AITD: autoimmune thyroid disease
HT: Hashimoto’s thyroiditis
EHT: experimental Hashimoto’s thyroiditis
MG: myasthenia gravis
NMJ: neuromuscular junction
AChR: acetylcholine receptor
MuSK: Muscle-Specific Kinase
AChR: anti-acetylcholine receptor
CTLA4Ig: CTLA4 immunoglobulin
